# Shock indices are associated with in-hospital mortality among patients with septic shock and normal left ventricular ejection fraction

**DOI:** 10.1371/journal.pone.0298617

**Published:** 2024-03-12

**Authors:** Kyu Jin Lee, Yong Kyun Kim, Kyeongman Jeon, Ryoung-Eun Ko, Gee Young Suh, Dong Kyu Oh, Sung Yoon Lim, Yeon Joo Lee, Su Yeon Lee, Mi-Hyeon Park, Chae-Man Lim, Sunghoon Park, on behalf of the Korean Sepsis Alliance (KSA) investigators

**Affiliations:** 1 Department of Pulmonary, Allergy and Critical Care Medicine, Hallym University Sacred Heart Hospital, Anyang, Republic of Korea; 2 Department of Infection, Hallym University Sacred Heart Hospital, Anyang, Republic of Korea; 3 Department of Critical Care Medicine, Samsung Medical Center, Sungkyunkwan University School of Medicine, Seoul, Republic of Korea; 4 Department of Pulmonary and Critical Care Medicine, Asan Medical Center, University of Ulsan College of Medicine, Seoul, Republic of Korea; 5 Department of Pulmonary and Critical Care Medicine, Seoul National University Bundang Hospital, Seongnam, Republic of Korea; Ajou University School of Medicine, REPUBLIC OF KOREA

## Abstract

**Background:**

The understanding of shock indices in patients with septic shock is limited, and their values may vary depending on cardiac function.

**Methods:**

This prospective cohort study was conducted across 20 university-affiliated hospitals (21 intensive care units [ICUs]). Adult patients (≥19 years) with septic shock admitted to the ICUs during a 29-month period were included. The shock index (SI), diastolic shock index (DSI), modified shock index (MSI), and age shock index (Age-SI) were calculated at sepsis recognition (time zero) and ICU admission. Left ventricular (LV) function was categorized as either normal LV ejection fraction (LVEF ≥ 50%) or decreased LVEF (<50%).

**Results:**

Among the 1,194 patients with septic shock, 392 (32.8%) who underwent echocardiography within 24 h of time zero were included in the final analysis (normal LVEF: n = 246; decreased LVEF: n = 146). In patients with normal LVEF, only survivors demonstrated significant improvement in SI, DSI, MSI, and Age-SI values from time zero to ICU admission; however, no notable improvements were found in all patients with decreased LVEF. The completion of vasopressor or fluid bundle components was significantly associated with improved indices in patients with normal LVEF, but not in those with decreased LVEF. In multivariable analysis, each of the four indices at ICU admission was significantly associated with in-hospital mortality (P < 0.05) among patients with normal LVEF; however, discrimination power was better in the indices for patients with lower lactate levels (≤ 4.0 mmol/L), compared to those with higher lactate levels.

**Conclusions:**

The SI, DSI, MSI, and Age-SI at ICU admission were significantly associated with in-hospital mortality in patients with septic shock and normal LVEF, which was not found in those with decreased LVEF. Our study emphasizes the importance of interpreting shock indices in the context of LV function in septic shock.

## Introduction

Sepsis, a life-threatening condition resulting from a dysregulated host response to infection, is a significant global health concern [1]. Despite advancements in understanding its underlying mechanisms, sepsis continues to be associated with high morbidity and mortality rates [2, 3].

During the early phase of circulatory shock, vital signs may not exhibit significant changes due to compensatory responses. To enhance hemodynamic assessment, the use of the shock index (SI), defined as the ratio of heart rate (HR) to systolic blood pressure (SBP), has been explored in various populations [4–7]. In addition, modifications of the SI, such as the modified shock index (MSI) [8], diastolic shock index (DSI) [9], and age shock index (Age-SI) [10], have been proposed for critically ill patients. These indices reflect the interaction between the heart and vasculature and offer the advantages of simplicity and rapid bedside assessment.

However, despite several studies, there are limited data on the application of these shock indices in patients with sepsis. The optimal timing and utility of these indices, particularly in vasopressor-dependent septic shock, remain unclear. Furthermore, considering the complex pathophysiology of sepsis and the presence of various confounding factors, limitations may exist in using these indices in sepsis or septic shock patients. Of particular relevance, their values may vary depending on left ventricular (LV) function. To date, only a few studies have investigated the association between shock indices and patient outcomes while considering LV function.

Therefore, in this study, our hypothesis was that the effects of SI, DSI, MSI, and Age-SI may vary based on the presence of left ventricular (LV) dysfunction in patients with septic shock. To explore this, we analyzed data from a prospective sepsis cohort conducted by the Korean Sepsis Alliance (KSA). We investigated potential associations between the shock indices and hospital outcomes specifically in patients with vasopressor-dependent septic shock.

## Methods and materials

### Study population

We collected data prospectively from 20 tertiary or university-affiliated hospitals, including 21 intensive care units (ICUs), as part of an ongoing nationwide multicenter cohort study led by the KSA. These hospitals are actively involved in sepsis bundle educational programs. To ensure data quality, regular audits were performed by research committee members, and each site received weekly feedback from the committee. For this particular study, we analyzed data collected over a period of 29 months, from August 2019 to December 2021. We screened consecutive patients (≥19 years old) who were diagnosed with sepsis or septic shock in the emergency departments (Eds) or general wards for eligibility. The inclusion criteria were patients with septic shock requiring vasopressors and with lactate levels > 2 mmol/L, patients admitted to the ICUs, and patients who underwent transthoracic echocardiography (TTE) within 24 h of sepsis recognition (referred to as time zero). Exclusion criteria included patients with sepsis alone, time intervals from time zero to ICU admission exceeding 24 h, missing data on vital signs or lactate, and initial heart rates below 60 or above 180 beats/min. We used the Sepsis-3 criteria for diagnosing sepsis and septic shock. The institutional review boards (IRB) of each participating hospital approved this study, which was conducted in accordance with the Helsinki Declaration of 1975, as most recently amended. Given the observational nature of the study, the decision to obtain written informed consent was left to the discretion of the ethics committees at each participating institution. We adhered to the STROBE guidelines for reporting observational cohort studies [11].

### Data collection

Trained operators prospectively collected data at each hospital and recorded the information in a web-based database system (http://sepsis.crf.kr/). The collected data included the following: demographic information (age, sex, and body mass index), comorbidities, physiological and laboratory parameters, Sequential Organ Failure Assessment (SOFA) score at sepsis recognition (referred to as “time zero”) and ICU admission, Simplified Acute Physiology Score 3 (SAPS3) at ICU admission, infection origins and types (community-acquired or hospital-acquired), presence of multidrug-resistant (MDR) pathogens, appropriateness of empirical antibiotic therapy, completion rates of the 3 h sepsis bundle components (lactate measurement, blood culture, antibiotics, fluids, and vasopressors), ICU treatments such as mechanical ventilation (MV) and continuous renal replacement therapy (CRRT) until ICU day 3, and ICU, 30-day, and in-hospital mortality rates.

The shock indices (SI, DSI, MSI, and Age-SI) were calculated both at time zero and at ICU admission for each patient. LV function was determined based on LV ejection fraction (LVEF). LVEF was predominantly assessed via visual estimation through two-dimensional imaging, incorporating fractional shortening in the parasternal long axis view, following the guidelines of the American Society of Echocardiography [12]. Although the data were initially categorized into subgroups based on LVEF (i.e., normal function [≥50%], mild dysfunction [40–49%], moderate dysfunction [20–39%], and severe dysfunction [<20%]), for the purpose of analysis, we divided patients into two groups: those with normal LVEF (≥50%) and those with decreased LVEF (<50%).

In patients who were diagnosed with sepsis at the ED, “time zero” was defined as the time of triage in the ED, and for those who were diagnosed with sepsis at general wards (during the hospitalization), its time zero was defined as when the rapid response team recognized sepsis in the general ward [13, 14]. The appropriateness of empirical antibiotics was determined according to the results of the drug susceptibility test or assessed according to the relevant guidelines [15, 16]. MDR organisms were defined as those resistant to antibiotics from at least three antimicrobial classes [17]. All information was processed anonymously.

### Definitions for shock indices

The systolic shock index (SI) was calculated by dividing heart rate (HR) by systolic blood pressure (SBP). Similarly, the diastolic shock index (DSI) was derived by dividing HR by diastolic blood pressure (DBP). The modified shock index (MSI) is another variation, computed as the HR divided by mean arterial pressure (MAP). Finally, the age shock index (Age-SI) was calculated as the SI multiplied by the patient’s age.

### Data analyses

Primary outcomes were the association between the SI, DSI, MSI, and Age-SI (both at time zero and at ICU admission) and in-hospital mortality rate. Secondary outcomes were changes of the indices according to the completion of 3-h bundle components, and the association between the indices and ICU and 30-day mortality rates.

Categorical variables are presented as numbers (%), and continuous variables as means ± standard deviations or medians with interquartile ranges (25% ~ 75%). To compare continuous variables, student t test or Mann-Whitney *U* test were used, and for categorical variables, the chi-square or Fisher’s exact test was used. To investigate the association between the shock indices and patients’ mortality rates, we performed multivariable logistic regression analyses using clinically relevant covariates with a P value of < 0.10 in univariable analyses; age variable was included in the final model because of its clinical significance. Receiver operating characteristics (ROC) curves were also plotted to investigate the discrimination power of the shock indices for predicting in-hospital mortality. For this analysis, patients were divided into two groups and analyzed separately, according to the lactate levels (i.e., > 4.0 vs. ≤ 4.0 mmol/L). Area under the RUC (AUC) values of < 0.7, 0.7 to 0.8, and 0.8 to 0.9, and > 0.9 were interpreted as low, moderate, good, and excellent discrimination power, respectively [18]. All tests were two-sided, and a P value of < 0.05 was considered to indicate statistical significance. IBM SPSS for Windows software (ver. 26.0; IBM Corp., Armonk, NY, USA) was used for all statistical analyses.

## Results

### Study population

**Fig 1:**
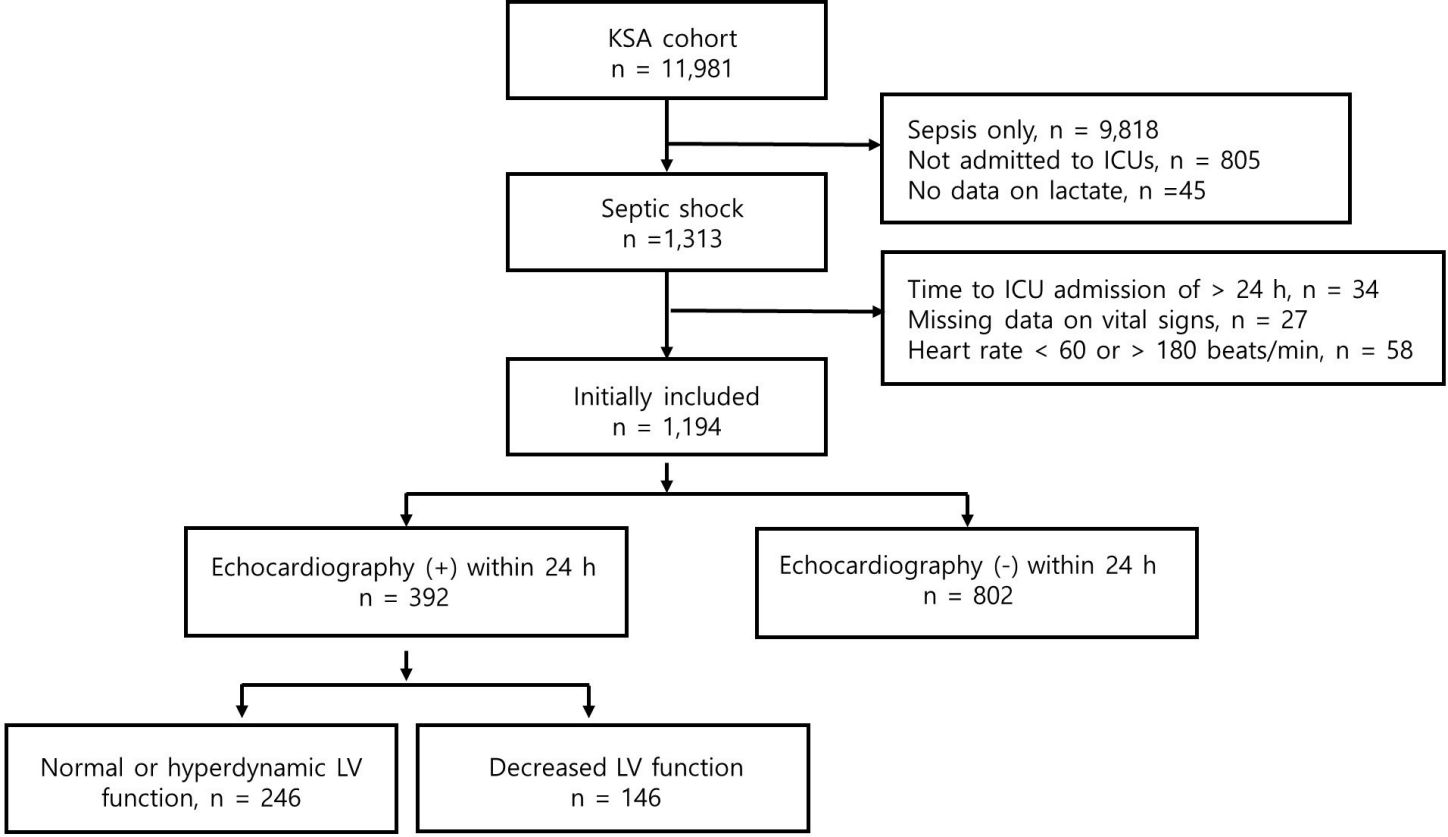
Flow chart for the enrolled patients.

During the study period, 11,981 patients with sepsis were registered. After excluding 10,787 patients, 1,194 patients with septic shock were initially included (**S1 Table**). Among them, 392 patients (32.8%) who underwent TTE within 24 h were ultimately included (**Fig 1**). The mean age of the included patients was 70.9 ± 13.5 years, and 42.3% were female (**Table 1**). The most common underlying comorbidities were diabetes mellitus (43.1%) and chronic heart disease (36.5%), while the most common sites of sepsis origin were the lung (36.0%) and abdomen (24.1%). However, comorbidities (i.e., chronic heart or lung disease) and pulmonary sepsis were more common in those with decreased LVEF, compared to those with normal LVEF. Besides, Charlson comorbidity index and illness severity, as well as lactate levels, were higher in the former group (**Tables 1** and **S2**).

**Table 1:** Baseline characteristics between patients with normal and decreased LVEF.

Variables	Total (n = 392)	Normal LVEF (n = 246)	Decreased LVEF (n = 146)	P
Age, years	70.9 ± 13.5	70.5 ± 14.5	71.6 ± 13.6	0.418
Gender, M/F	226/166	134/112	92/54	0.098
Body mass index, kg/m^2^	22.3 ± 3.9	23.6 ± 4.3	21.7± 3.4	**0.020**
Comorbidities				
Chronic heart disease	143 (36.5%)	79 (32.1%)	64 (43.8%)	**0.020**
Chronic lung disease	85 (21.7%)	45 (18.3%)	40 (27.4%)	**0.024**
Central nervous system	101 (25.9%)	61 (24.8%)	40 (27.4%)	0.569
Chronic liver disease	45 (11.5%)	29 (11.8%)	16 (11.0%)	0.803
Diabetes	169 (43.1%)	100 (40.7%)	69 (47.3%)	0.201
Chronic kidney disease	71 (18.1%)	45 (18.3%)	26 (17.8%)	0.904
Connective tissue disease	12 (3.1%)	10 (4.1%)	2 (1.4%)	0.134
Immunocompromised	8 (2.0%)	5 (2.0%)	3 (2.1%)	0.988
Cancer	110 (28.1%)	65 (26.4%)	45 (30.8%)	0.349
Sepsis origins				
Pulmonary	141 (36.0%)	73 (29.7%)	68 (46.6%)	**0.010**
Abdominal	95 (24.1%)	70 (28.5%)	25 (17.1%)	
Urinary	85 (21.7%)	58 (23.6%)	27 (18.5%)	
Skin and soft tissue	23 (5.9%)	16 (2.5%)	7 (4.8%)	
Central nervous system	2 (0.5%)	2 (0.8%)	0 (0.0%)	
Catheter	1 (0.3%)	0 (0.0%)	1 (0.7%)	
Unclear origins	45 (11.5%)	27 (11.0%)	18 (12.3%)	
Charlson comorbidity index	5.6 ± 2.8	5.2 ±2.6	6.1 ± 3.2	**0.005**
SOFA at time zero	9.2 ± 3.3	8.8 ± 3.2	9.9 ± 3.3	**0.001**
SOFA at ICU admission	11.2 ± 3.3	10.3 ±3.7	11.3 ± 4.0	**0.010**
SAPS3 at ICU admission	80.1 ± 15.7	77.0 ± 14.9	85.1 ±15.7	**< 0.001**
CAI/HAI	252/140	144/102	108/38	**0.002**
Pathogen-proven	274 (69.9%)	168 (68.3%)	106 (72.6%)	
Bacterial origin	252 (92.0%)	153 (91.1%)	99 (93.4%)	0.218
G(+)/G(-) ^a^	65/187	35/118	30/69	0.220
MDR pathogens ^a^	65 (25.8%)	35 (22.9%)	30 (30.3%)	0.188
Bacteremia	157 (40.1%)	101 (41.1%)	56 (38.4%)	0.598

CAI, community-acquired infection; F, female; G (+), gram-positive organism; G (-), gram-negative organism; HAI, hospital-acquired infection; LVEF, left ventricular ejection fraction (normal LVEF, ≥ 50%; decreased LVEF, < 50%); M, male; MDR, multi-drug resistance; SAPS, simplified acute physiology score; SOFA, sequential organ failure assessment.

^a^ Among those with bacterial origins

The completion rates of the 3 h sepsis bundle components were 71.2% for antibiotics, 84.7% for fluids, and 86.5% for vasopressors among all enrolled patients (**S3 Table**). Mechanical ventilation (MV) and continuous renal replacement therapy (CRRT) were performed more frequently in patients with decreased LVEF than in those with normal LVEF.

### Comparisons of the shock indices values between survivors and non-survivors

**Fig 2:**
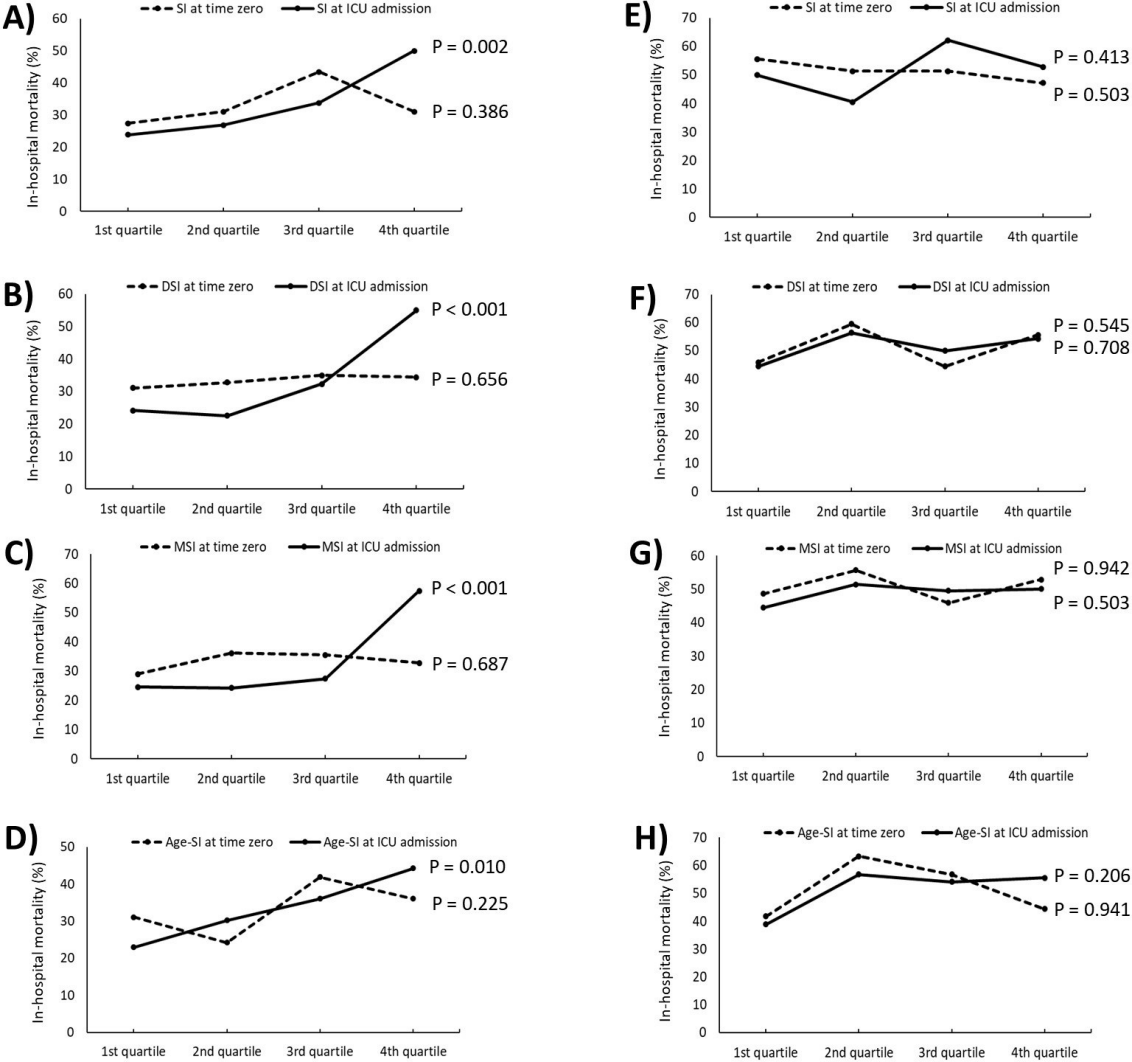
In-hospital mortality rates across the quartiles for SI, DSI, MSI, and Age-SI values among patients with normal LVEF (≥ 50%; A, B, C, and D, respectively) and those with decreased LVEF (< 50%; E, F, G, and H, respectively). SI, shock index; DSI, diastolic shock index; MSI, modified shock index; Age-SI, age shock index; LVEF, left ventricular ejection fraction.

The in-hospital, ICU, and 30-day mortality rates were 40.1%, 31.4%, and 33.2%, respectively. Among those with an SI > 1.0, in-hospital mortality rates at both time zero and ICU admission were 40.9% and 45.7%, respectively, and when segmented by LVEF, the mortality rate increased as the LVEF decreased (**S1 Fig)**. For patients with normal LVEF, all shock indices at ICU admission were notably lower in survivors than in non-survivors (**Table 2**). Besides, the in-hospital mortality rates showed a significant increasing tendency across the quartiles for each of the four indices (for all the indices, P < 0.05 by chi-square test for trend; **Fig 2**).

**Table 2:** Vital signs and shock indices between survivors and non-survivors stratified by LVEF (n = 392).

	Normal LVEF ^a^				Decreased LVEF ^b^	
Variables	Survivors (n = 164)	Non-survivors (n = 82)	P	Survivors (n = 71)	Non-survivors (n = 75)	P
**At time zero**						
Systolic BP, mm Hg	87.9 ± 27.2	84.6 ± 25.1	0.366	85.7 ± 24.8	84.0 ± 23.6	0.684
Diastolic BP, mm Hg	53.3 ± 18.3	52.1 ± 17.7	0.635	53.2 ±16.9	50.5 ± 16.4	0.329
Heart rate, beats/min	107.5 ± 24.5	107.5 ± 21.0	0.997	113.1 ± 24.4	110.4 ± 23.8	0.502
Respiratory rate, /min	23.9 ± 6.8	24.1 ± 6.9	0.906	26.2 ± 7.4	25.9 ± 6.2	0.824
BT, °C	37.1 ± 1.2	36.9 ± 1.1	**0.012** ^**c**^	37.3 ±1.2	37.3 ± 1.1	0.918
SI	1.3 ± 0.5	1.3 ± 0.4	0.827	1.4 ± 0.4	1.4 ± 0.5	0.928
MSI	1.8 ± 0.7	1.8 ± 0.5	0.863	1.9 ± 0.6	1.9 ± 0.8	0.622
DSI	2.2 ± 0.9	2.3 ± 0.9	0.722	2.3 ± 0.8	2.5 ±1.1	0.314
Age-SI	94.2 ± 45.6	95.2 ± 35.5	0.853	98.6 ± 35.8	102.4 ± 42.2	0.554
Lactate, mmol/L	4.1 (2.8–6.3)	5.0 (2.9–9.6)	**0.002**	5.2 (3.6–7.4)	6.1 (3.9–7.9)	0.269
**At ICU admission**						
Systolic BP, mm Hg	107.1 ± 24.3 ^d^	99.3 ± 29.1 ^d^	**0.027** ^**c**^	97.1 ± 28.2 ^d^	94.2 ± 26.2 ^d^	0.525
Diastolic BP, mm Hg	61.2 ± 15.1 ^d^	55.2 ±14.8	**0.003** ^**c**^	56.8 ±17.9	54.8 ± 16.9	0.480
Heart rate, beats/min	106.2 ± 25.3	113.4 ± 27.1 ^d^	**0.042** ^**c**^	117.1 ± 26.2	121.1 ± 26.5 ^d^	0.368
Respiratory rate, /min	23.5 ± 5.9	23.7 ± 6.9	0.796	26.2 ± 6.3	25.0 ± 7.8	0.320
BT, °C	37.1 ± 0.9 ^d^	36.9 ± 1.2	0.157	37.4 ±1.2	37.4 ± 1.3	0.956
SI	1.0 ± 0.4 ^d^	1.2 ± 0.5	**0.001** ^**c**^	1.3 ± 0.6	1.4 ± 0.6	0.456
MSI	1.5 ± 0.5 ^d^	1.7 ±0.6	**< 0.001** ^**c**^	1.9 ± 0.8	1.9 ± 0.8	0.569
DSI	1.8 ± 0.6 ^d^	2.2 ± 0.7	**< 0.001** ^**c**^	2.4 ± 1.2	2.5 ± 1.1	0.587
Age-SI	73.6 ± 31.9 ^d^	85.3 ± 35.2 ^d^	**0.009** ^**c**^	93.9 ± 45.3	102.1 ± 50.7	0.310
Lactate, mmol/L	3.6 (2.4–5.8)^d^^d^	4.9 (2.9–8.8)	**0.001**	5.0 (2.9–6.9) ^d^	5.9 (4.2–8.6)	**0.016**

Age-SI, age shock index; BT, body temperature; BP, blood pressure; DSI, diastolic shock index; ICU, intensive care unit; LVEF, left ventricular ejection fraction MSI, modified shock index; SI, systolic shock index.

^a^ LVEF ≥ 50%, ^b^ LVEF < 50%. ^c^ P < 0.05 between survivors and non-survivors,

^d^ P < 0.05 by paired tests between time zero and ICU admission.

### Changes in the shock indices from time zero to ICU admission

**Fig 3:**
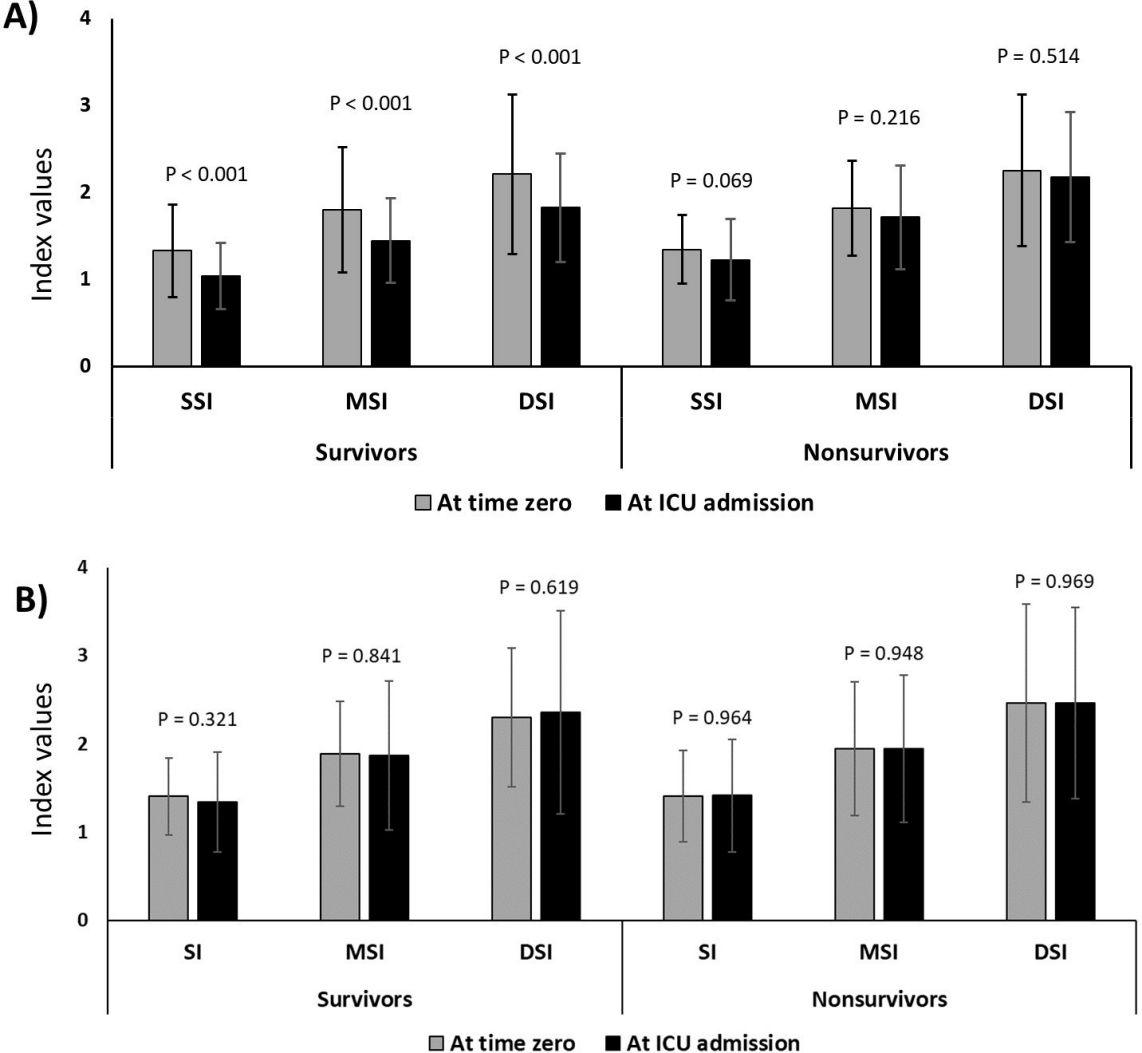
Differnces in the SI, DSI, and MSI values between time zero and ICU admission; A) patients with normal LVEF (≥ 50%) and B) patients with decreased LVEF (< 50%). SI, shock index; DSI, diastolic shock index; MSI, modified shock index; LVEF, left ventricular ejection fraction. Data on Age-SI are presented in **S2 Fig**.

Significant improvements (i.e., decreased values) in the SI, DSI, MSI, and Age-SI values from time zero to ICU admission were found in survivors with normal LVEF. However, no considerable changes were observed in non-survivors or in those with reduced LVEF (**Figs 3 and S2**). As for the components of the 3 h sepsis bundle, successful completion of the vasopressor component was significantly associated with improved indices (for all the indices, P < 0.05) in patients with normal LVEF; completion of the fluid component was also associated with improved indices (**S4 Table**).

### Multivariable analysis for in-hospital mortality in patients with normal LVEF

Because of nonsignificant differences in patients with decreased LVEF, we only performed mutivariable analyses in those with normal LVEF. We controlled for age, body temperature, cancer, SOFA, SAPS3, hospital-acquired infection, antibiotic adequacy, and MV and CRRT treatments in the models; except for age, all variables had a P < 0.1 in univariable analyses (**S5 Table**). In the final models, all four indices at ICU admission and their respective quartiles (except for the Age-SI quartile) were significantly associated with increased in-hospital mortality (**Table 3**).

**Table 3:** Multivariable analyses for hospital outcomes by logistic regression models among patients with normal LVEF (EF ≥ 50%) ^a^.

Shock indices at ICU admission	Hospital mortality		ICU mortality		30-day mortality	
	Ors (95% Cis)	P value	Ors (95% Cis)	P value	Ors (95% Cis)	P value
**Vital signs**						
Systolic BP	0.985 (0.973 to 0.998)	**0.027** ^b^	0.985 (0.971 to 0.999)	**0.032** ^b^	0.985 (0.971 to 0.999)	**0.033** ^b^
Diastolic BP	0.973 (0.950 to 0.997)	**0.027** ^b^	0.975 (0.950 to 1.000)	0.054	0.980 (0.955 to 1.005)	0.114
Heart rate	1.009 (0.955 to 1.022)	**0.207**	1.003 (0.988 to 1.017)	0.713	1.008 (0.994 to 1.022)	0.279
**Shock indices**						
SI	2.953 (1.271 to 6.861)	**0.012** ^b^	2.192 (0.912 to 5.265)	0.079	2.461 (1.026 to 5.900)	**0.044** ^b^
DSI	2.010 (1.194 to 3.386)	**0.009** ^b^	1.586 (0.916 to 2.746)	0.100	1.607 (0.928 to 2.780)	0.079
MSI	2.482 (1.284 to 4.797)	**0.007** ^b^	1.845 (0.926 to 3.677)	0.087	1.985 (0.996 to 3.955)	0.051
Age SI	1.014 (1.003 to 1.025)	**0.012** ^b^	1.011 (1.000 to 1.023)	0.055	1.013 (1.002 to 1.025)	**0.025** ^b^
SI quartiles	1.435 (1.051 to 1.959)	**0.023** ^b^	1.364 (0.972 to 1.913)	0.072	1.408 (1.004 to 1.975)	**0.047** ^b^
DSI quartiles	1.475 (1.089 to 1.998)	**0.012** ^b^	1.243 (0.896 to 1.726)	0.193	1.434 (1.030 to 1.997)	**0.033** ^b^
MSI quartiles	1.511 (1.111 to 2.056)	**0.009** ^b^	1.404 (1.006 to 1.959)	**0.046** ^b^	1.427 (1.023 to 1.990)	**0.036** ^b^
Age-SI quartiles	1.367 (0.991 to 1.911)	0.056	1.241 (0.876 to 1.756)	0.224	1.323 (0.933 to 1.877)	0.117

Age-SI, age shock index; BP, blood pressure; CI, confidence interval; DSI, diastolic shock index; LVEF, left ventricular ejection fraction; MSI, modified shock index; OR, odds ratio; SI, systolic shock index.

^a^ Adjusted for age, body temperature, cancer, SOFA score and SAPS3 at ICU admission, hospital-acquired infection, mechanical ventilation, continuous renal replacement therapy, and antibiotic adequacy.

^b^ P < 0.05

### ROC curves for prediciting in-hospital mortality in patients with normal LVEF

**Fig 4:**
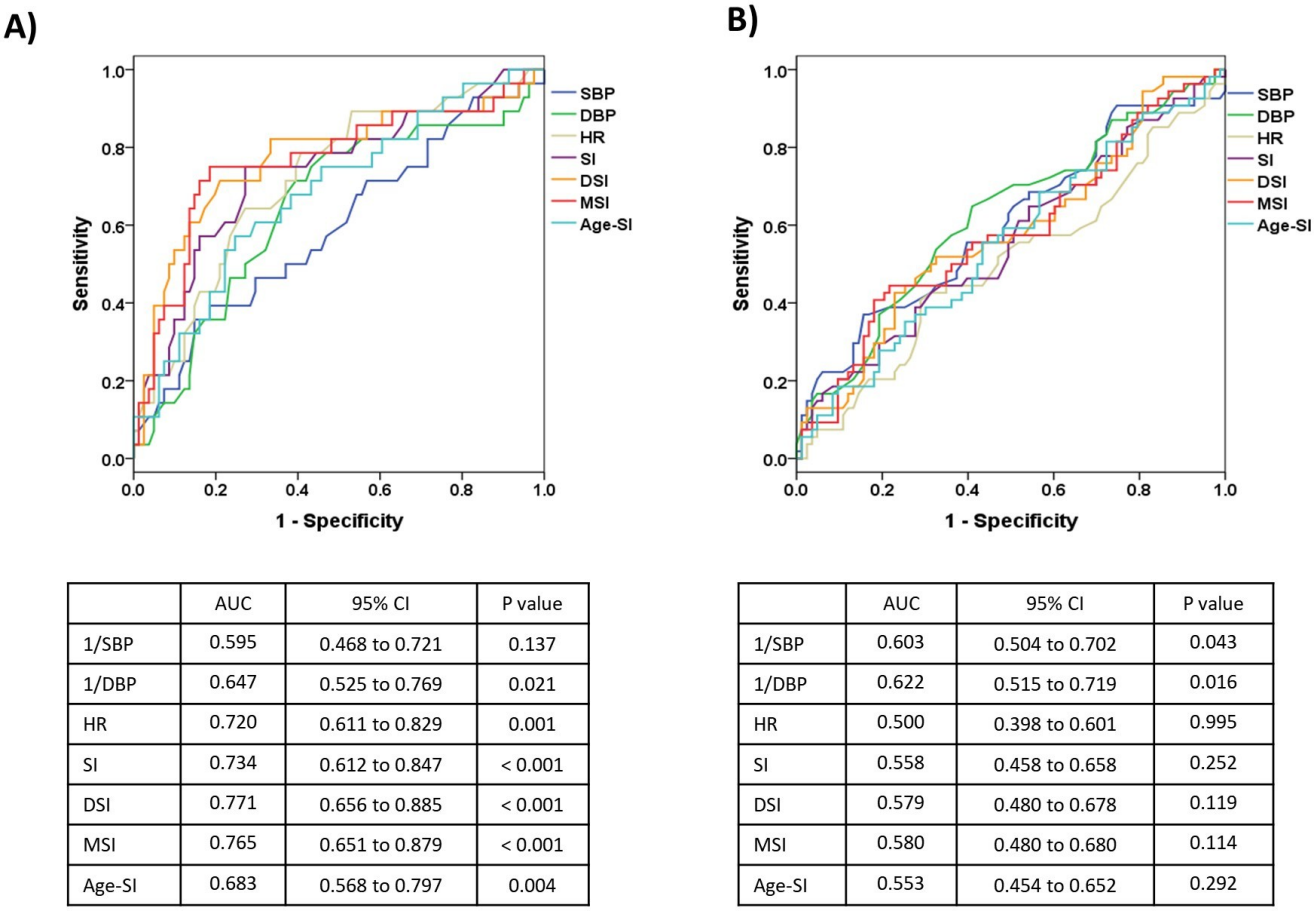
Receiver operating characteristics (ROC) curves for predicting in-hospital mortality among 262 patients with normal LVEF (≥ 50%): A) ROC curves for patients with lactate levels of ≤ 4.0 mmol/L (n = 109) and B) ROC curves for patients with lactate levels of > 4.0 mmol/L (n = 137). AUC, area under the ROC curve; CI, confidence interval; SBP, systolic blood pressure; DBP, diastolic blood pressure; HR, heart rate; SI, shock index; DSI, diastolic shock index; MSI, modified shock index; Age-SI, age shock index; LVEF, left ventricular ejection fraction.

Among patients with normal LVEF, the ROC curves of the shock indices displayed low discriminatory capability for predicting in-hospital mortality (**S3 Fig**). However, once segmented by a clinically important lactate level (i.e., 4.0 mmol/L), better discrimination power was observed in the indices for patients with lactate levels of ≤ 4.0 mmol/L (n = 109), compared to those with lactate levels of > 4.0 mmol/L (n = 137). The areas under the ROC curves for the SI, DSI, and MSI were 0.734, 0.771, and 0.765, respectively in the former group (**Fig 4A and 4B**).

## Discussion

In this prospective, multicenter cohort, we noted several intriguing findings. First, the SI, DSI, MSI, and Age-SI values at ICU admission, as opposed to those at time zero (i.e., at the point of sepsis recognition), proved to be more predictive of hospital outcomes. This significant correlation was observed in patients with normal LVEF but not in those with decreased LVEF. Second, the successful completion of either the vasopressor or fluid bundle components was linked with decreased (improved) values in the shock indices in patients with normal LVEF. Finally, the efficacy of these shock indices for predicting in-hospital mortality was better among those with lower lactate levels (<4.0 mmol/L), compared to those with higher lactate levels.

Shock indices offer approximate measurements of hemodynamic status in critically ill patients. While some results remain equivocal, both the SI (> 0.9) and MSI (> 1.3) have been demonstrated to predict hemorrhagic shock or mortality in trauma patients [19–22]. In the case of patients with ST-elevation myocardial infarction, an SI ≥ 0.8 upon admission is associated with increased mortality (20.3% vs. 4.0% in those with an SI < 0.8) [23]. For patients with pulmonary thromboembolism, an SI ≥ 1.0 is associated with right ventricular dysfunction and increased hospital mortality [24].

In terms of sepsis patients, a retrospective study involving 2,524 adult participants demonstrated that an SI ≥ 0.7 possessed a high negative predictive value (NPV) for hyperlactatemia (NPV, 95%) and 28-day mortality (NPV, 89%) [25]. A prospective observational cohort of 25 patients with septic shock indicated that patients with an SI ≤ 1 and central venous pressure ≥ 8 mm Hg were unlikely to respond to volume expansion [26]. In another investigation of 295 patients with severe sepsis, a higher number of patients with sustained SI values > 0.8 required vasopressor treatments compared to those without a sustained increase in SI [27]. However, in the current study, in-hospital mortality rates in patients with an SI > 1.0 at both time zero and ICU admission were 40.9% and 45.7%, respectively (**S6 Table**). These rates seem higher than those reported in prior research (SI > 1.0: 28.3% in trauma [28], 23.3% in severe sepsis [25], 22.2% in pulmonary thromboembolism [24], and <20% in ST-elevation myocardial infarction [23]). This discrepancy may be partially explained by different pathophysiological or hemodynamic conditions across studies. It is worth noting that our study population consisted of conditions where vasoplegia was predominant, a scenario that contrasts with previous studies.

One of the limitations of our study was the initial exclusion of a large proportion of patients. However, as previously mentioned, sepsis has a complex pathophysiology and is influenced by various confounding factors. To bolster the validity of our results, we limited our inclusion to patients who had TTE data collected within 24 h of time zero. Tachycardia may indicate grave outcomes in patients with predominantly vasoplegic shock, but it can be a compensatory response to increase cardiac output in those with decreased LVEF (e.g., septic or stress-induced cardiomyopathy) [29–31]. Therefore, considering cardiac function when evaluating the effects of vital signs or their surrogates is paramount. In this regard, our findings have merit and could provide guidance for the utilization of shock indices in septic shock.

Recently, Ospina-Tascon *et al*. studied the usefulness of DSI in patients with septic shock, using two cohorts (a preliminary cohort and a randomized controlled trial) [9]. They reported a progressively increasing risk of death with a gradual uptick in DSI, and that the interaction between DSI and norepinephrine dose was significantly higher in nonsurvivors than in survivors [9]. Regrettably, in our study, only vital signs at two time points (i.e., at time zero and ICU admission) were collected, and data on the types and doses of vasopressors were not available, which is one of the major limitations of our study. However, despite this, we found that the four shock indices measured at ICU admission, rather than those measured at time zero, were beneficial for predicting hospital outcomes. This result partly aligns with previous findings that suggested that persistent tachycardia following fluid or vasopressor therapies may be detrimental [32, 33].

Like other scores derived from vital signs [34], one might consider that these shock indices are more appropriate for screening or identifying specific patient groups requiring urgent interventions rather than predicting hospital outcomes. Indeed, the multitude of confounding factors influencing both vital signs and patient outcomes during an ICU stay can make these indices challenging to utilize. Nevertheless, in the current study, after adjusting for various covariates such as illness severity, organ failure score, antibiotic adequacy, and ICU treatments, the shock indices at ICU admission were significantly associated with in-hospital mortality in patients with normal LVEF. Particularly, given the results of the ROC curves, the shock indices may perform better in patients with lower lactate levels. Collectively, our results suggest that shock indices may be more beneficial for patients with normal LV function and less severe vasoplegia, rather than for those with decreased LV function or severe vasoplegia. Although the exact mechanism for this result is unclear, we found that the shock indices were only improved in the survivors with normal LVEF, not in survivors with decreased LVEF (Table 2). As opposed to lactate levels which were improved at ICU admission, no distinct changes were noted in the shock indices in the survivors with decreased LVEF. Hence, our results may suggest that the shock indices do not reflect well on the changes of hemodynamic status or tissue perfusion in patients with decreased LVEF. Besides, given the higher comorbidity index and illness severity in patients with decreased LVEF than in those with normal LVEF, many confounding factors during the sepsis management may have influenced the association between the shock indices and in-hospital mortality in the former group. These factors may partly explain why the shock indices were not associated with in-hospital mortality in those with decreased LVEF or hyperlactatemia. However, due to the small sample size of our study, these findings should be confirmed through further large-scale studies.

Interestingly, changes in the shock indices following the completion of the 3 h vasopressor or fluid bundle components were more pronounced in patients with normal LVEF than in those with decreased LVEF. This might imply that treatment responses during the early phase of sepsis can be evaluated by changes in the shock indices in patients whose LV function is preserved. Regrettably, we were unable to differentiate between hyperdynamic and normodynamic LVEF, a distinction for which Paonessa *et al*. reported an increased 28-day mortality in patients with hyperdynamic LVEF, compared to those with normodynamic LVEF [30]. Thus, further studies are needed to better elucidate any differences in outcomes between these two groups.

Several limitations should be noted. First, due to its observational nature and the exclusion of a large proportion of patients, our results may lack statistical power and be subject to selection bias. Second, the precise time intervals (h) from time zero to ICU admission were not available in our study. Although it is reasonable to assume that most sepsis patients underwent initial resuscitation prior to ICU admission, the timing of ICU admission can vary depending on the hospital. In some hospitals, patients are admitted to the ICU after the completion of resuscitation, while in others they are in the process of resuscitation at the time of ICU admission. Third, we did not obtain detailed echocardiographic findings, nor did we differentiate hyperdynamic from normodynamic LVEF. Notably, the presence of diastolic heart failure or ventricular-arterial uncoupling may have influenced changes in HR or cardiac contractility [35, 36]. Besides, because no specific protocols for echocardiography were used, the decision to obtain echocardiography was at the discretion of physicians, and different management strategies were used. Fourth, although it is still not fully understood, higher levels of cytokines may impact cardiac contractility in sepsis [31], a consideration that was beyond the scope of our investigation. Fifth, we were unable to identify and exclude patients with arrhythmia. However, to mitigate this possibility, we excluded patients with an HR of less than 60 or more than 180 beats per minute. Sixth, because shock can be compensated by vasopressors, doses of vasopressors should be taken into account when evaluating the usefulness of shock indicies. However, the data were not available in our cohort. Finally, as this study was conducted in a single country, the generalizability of our findings may be limited. Besides, the variable selection method used for multivariable analyses (post-hoc covariate selection) in this study may also impede the generalizability. However, few studies to date have examined shock indices in septic shock or have incorporated LV function into the analysis. Our study may provide some insight into the use of shock indices in patients with septic shock. Future large-scale studies are required to corroborate our results.

## Conclusions

The SI, DSI, MSI, and Age-SI at ICU admission were significantly associated with in-hospital mortality in patients with septic shock and normal LVEF. Our study suggests that evaluating shock indices after early sepsis resuscitation, rather than at time zero, may be more valuable in cases of septic shock. Specifically, our findings indicate that this approach is particularly beneficial for patients with normal LV function and lower lactate levels.

## Supporting information

S1 Table Characteristics of participating hospitals (1,194 patients with septic shock and 20 hospitals). TTE, transthoracic echocardiography.
(DOCX)

S2 Table Initial laboratory parameters between patients with normal LVEF and those with decreased LVEF. BUN, blood urea nitrogen; CRP, C-reactive protein; HR, heart rate; INR, international normalized ratio; LVEF, left ventricular ejection fraction (normal LVEF, ≥ 50%; decreased LVEF, < 50%); WBC, white blood cells. ^a^ Lactate values at ICU admission.
(DOCX)

S3 Table Completion rates of 3-h sepsis bundle components and ICU treatments between survivors and non-survivors, stratified by LVEF. CRRT, continuous renal replacement therapy; HFNC, high flow nasal cannula; LVEF, left ventricular ejection fraction (normal LVEF, ≥ 50%; decreased LVEF, < 50%); MV, mechanical ventilation; NIV, non-invasive ventilation.
(DOCX)

S4 Table Changes in the four shock indices both in patients who completed 3-h sepsis bundle components and in those who did not. Age-SI, age shock index; DSI, diastolic shock index; LVEF, left ventricular ejection fraction (normal LVEF, ≥ 50%; decreased LVEF, < 50%); MSI, modified shock index; SI, systolic shock index. ^a^ Delta values mean changes from the time zero to ICU admission: negative values indicate a decrease (i.e., improvement) in the shock indices at ICU admission compared to those at time zero. ^b^ N = 3.
(DOCX)

S5 Table Univariable analyses of risk factors for in-hospital mortality in patients with normal LVEF (N = 246). Age-SI, age shock index; CI, confidence interval; DSI, diastolic shock index; I, input; ICU, intensive care unit; LVEF, left ventricular ejection fraction (normal LVEF, ≥ 50%; decreased LVEF, < 50%); MSI, modified shock index; O, output; SAPS, simplified acute physiology score; SI, systolic shock index; SOFA, sequential organ failure assessment. ^a^ Within 3 h of the time zero.
(DOCX)

S6 Table Proportions of patients with SI > 1.0 by LVEF, lactate levels, and in-hospital mortality. ICU, intensive care unit; LVEF, left ventricular ejection fraction (normal LVEF, ≥ 50%; decreased LVEF, < 50%); SI, shock index.
(DOCX)

S1 Fig In-hospital mortality according to left ventricular ejection fraction (LVEF) among patients with septic shock. Normal function indicates LVEF of ≥ 50%, and mild, moderate, and severe dysfunctions indicate LVEF of 40–49%, 20–39%, and < 20%, respectively.
(DOCX)

S2 Fig Changes in Age-SI from time zero to ICU admission. A) patients with normal or hyperdynamic LVEF, B) patients with decreased LVEF. Age-SI, age shock index, LVEF, left ventricular ejection fraction.
(DOCX)

S3 Fig Receiver operating characteristics (ROC) curves for predicting in-hospital mortality among 262 patients with normal LVEF (≥ 50%). AUC, area under the ROC curve; CI, condifence interval; SBP, systolic blood pressure; DBP, diastolic blood pressure; HR, heart rate; SI, shock index; DSI, diastolic shock index; MSI, modified shock index; Age-SI, age shock index; LVEF, left ventricular ejection fraction.
(DOCX)
