# Supplementary material for: Shock indices are associated with in-hospital mortality among patients with septic shock and normal left ventricular ejection fraction
Source: PLoS One. 2024 Mar 12;19(3):e0298617. doi: 10.1371/journal.pone.0298617 (PMC10931483; doi:10.1371/journal.pone.0298617)
Supplement: S1 Table — TTE, transthoracic echocardiography. (DOCX) [file pone.0298617.s001.docx]

**S1 Table. Characteristics of participating hospitals (1,194 patients with septic shock and 20 hospitals).**

| Hospitals | Size of hospitals (beds) | Enrolled patients with septic shock | Completion rates of 3-h sepsis bundle components | | | | | Enrolled patients with TTE (+) |
| --- | --- | --- | --- | --- | --- | --- | --- | --- |
|  |  |  | Lactate  n (%) | Blood culture  n (%) | Antibiotics  n (%) | Fluids  n (%) | Vasopressors  n (%) |  |
| 1 | 692 | 19 | 18 (94.7) | 17 (89.5) | 12 (63.2) | 17 (89.5) | 13 (68.4) | 7 (36.8) |
| 2 | 1,048 | 30 | 29 (96.7 | 27 (90.0) | 14 (46.7) | 22 (73.3) | 23 (76.7) | 17 (56.7) |
| 3 | 874 | 49 | 49 (100.0) | 48 (98.0) | 28 (57.1) | 49 (100.0) | 47 (95.9) | 36 (73.5) |
| 4 | 1,309 | 44 | 44 (100.0) | 21 (47.7) | 35 (79.5) | 34 (77.3) | 44 (100.0) | 2 (4.5) |
| 5 | 1,985 | 105 | 100 (95.2) | 85 (81.0) | 77 (73.3) | 98 (93.3) | 84 (80.0) | 27 (25.7) |
| 6 | 521 | 29 | 29 (100.0) | 28 (96.6) | 23 (79.3) | 21 (72.4) | 29 (100.0) | 0 (0.0) |
| 7 | 1,763 | 61 | 61 (100.0) | 56 (91.8) | 47 (77.0) | 57 (93.4) | 59 (96.7) | 11 (18.0) |
| 8 | 2,732 | 242 | 240 (99.2) | 218 (90.1) | 199 (82.2) | 208 (86.0) | 240 (99.2) | 44 (18.1) |
| 9 | 2,426 | 2 | 2 (100.0) | 2 (100.0) | 2 (100.0) | 2 (100.0) | 2 (100.0) | 1 (50.0) |
| 10 | 500 | 15 | 14 (93.3) | 15 (100.0) | 9 (60.0) | 14 (93.3) | 14 (93.3) | 10 (66.7) |
| 11 | 1,205 | 54 | 53 (98.1) | 4 (7.4) | 38 (70.4) | 47 (87.0) | 53 (98.1) | 2 (3.7) |
| 12 | 1,001 | 6 | 5 (83.3) | 5 (83.3) | 1 (16.7) | 2 (33.3) | 6 (100.0) | 0 (0.0) |
| 13 | 998 | 156 | 146 (93.6) | 131 (84.0) | 103 (66.0) | 123 (78.8) | 96 (61.5) | 57 (36.5) |
| 14 | 631 | 4 | 4 (100) | 3 (75.0) | 1 (25.0) | 4 (100.0) | 4 (100.0) | 0 (0.0) |
| 15 | 1,078 | 92 | 91 (98.9) | 67 (72.8) | 66 (71.7) | 75 (81.5) | 82 (89.1) | 65 (70.7) |
| 16 | 1,139 | 123 | 123 (100.0) | 0 (0.0) | 91 (74.0) | 105 (85.4) | 123 (100.0) | 51 (41.5) |
| 17 | 658 | 26 | 25 (96.2) | 24 (92.3) | 19 (73.1) | 22 (84.6) | 26 (100.0) | 1 (3.8) |
| 18 | 1,303 | 72 | 70 (97.2) | 64 (88.9) | 57 (79.2) | 63 (87.5) | 66 (91.7) | 38 (52.8) |
| 19 | 842 | 49 | 48 (98/0) | 48 (98.0) | 41 (83.7) | 49 (100.0) | 48 (98.0) | 22 (44.9) |
| 20 | 843 | 16 | 16 (100.0) | 14 (87.5) | 7 (43.8) | 15 (93.8) | 15 (93.8) | 1 (6.3) |
| Total | 23,548 | 1,194 | 1,167 (97.7) | 877 (73.5) | 870 (72.9) | 1,027 (86.0) | 1,074 (89.9) | 392 (32.8) |

TTE, transthoracic echocardiography
