# Supplementary material for: Shock indices are associated with in-hospital mortality among patients with septic shock and normal left ventricular ejection fraction
Source: PLoS One. 2024 Mar 12;19(3):e0298617. doi: 10.1371/journal.pone.0298617 (PMC10931483; doi:10.1371/journal.pone.0298617)
Supplement: S2 Table — BUN, blood urea nitrogen; CRP, C-reactive protein; HR, heart rate; INR, international normalized ratio; LVEF, left ventricular ejection fraction (normal LVEF, ≥ 50%; decreased LVEF, < 50%); WBC, white blood cells. a Lactate values at ICU admission. (DOCX) [file pone.0298617.s002.docx]

**S2 Table. Initial laboratory parameters between patients with normal LVEF and those with decreased LVEF.**

| Variables | Total  (n = 392) | Normal LVEF  (n = 246) | Decreased LVEF  (n = 146) | *P* |
| --- | --- | --- | --- | --- |
| WBC, ⅹ10^3^/µL | 12.5 (6.3 to 18.7) | 13.7 (6.1 to 20.0) | 12.0 (6.9 to 16.5) | 0.407 |
| Hb, g/dL | 10.4 ± 2.4 | 10.5 ± 2.5 | 10.3 ± 2.4 | 0.317 |
| Platelet, ⅹ10^3^/µL | 140.0 (71.3 to 221.3) | 129.5 (66.5 to 218.3) | 158.0 (83.0 to 236.3) | 0.186 |
| INR | 1.49 (1.2 to 1.6) | 1.3 (1.2 to 1.6) | 1.4 (1.2 to 1.8) | 0.425 |
| CRP, mg/dL | 12.6 (6.1 to 20.4) | 13.2 (5.9 to 20.4) | 12.3 (6.7 to 20.3) | 0.976 |
| Lactate, mmol/L | 4.8 (3.1 to 7.4) | 4.4 (2.8 to 7.0) | 5.3 (3.6 to 7.8) | **0.004** |
| Lactate (ICU), mmol/L ^a^ | 4.6 (2.6 to 7.0) | 3.9 (2.5 to 6.5) | 5.4 (3.6 to 7.8) | **0.001** |
| Troponin I, ng/mL ^b^ | 0.07 (0.03 to 0.24) | 0.06 (0.02 to 0.13) | 0.16 (0.04 to 1.14) | **< 0.001** |
| BUN, mg/dL | 34.0 (22.0 to 53.0) | 34.0 (22.8 to 51.7) | 34.8 (20.2 to 53.1) | 0.719 |
| Creatinine, mg/dL | 1.7 (1.1 to 2.9) | 1.7 (1.0 to 2.9) | 1.5 (1.1 to 2.9) | 0.590 |
| Bilirubin, mg/dL | 0.9 (0.5 to 1.7) | 0.8 (0.5 to 1.7) | 0.9 (0.5 to 1.7) | 0.473 |
| Albumin, g/dL | 2.8 ± 0.6 | 2.7 ± 0.6 | 2.8 ± 0.6 | 0.167 |
| pH | 7.33± 0.14 | 7.35 ± 0.14 | 7.30 ± 0.15 | **0.002** |
| [HCO3^-^], mEq/L | 17.7 ± 6.1 | 17.9 ± 5.8 | 17.4 ± 6.6 | 0.423 |
| P_a_CO_2_ mm Hg | 33.1 ± 14.9 | 31.1 ± 11.3 | 36.5 ± 19.1 | **0.002** |
| P_a_O_2_, mm Hg | 96.8 ± 57.1 | 100.5 ± 59.2 | 90.8 ± 53.2 | 0.109 |

BUN, blood urea nitrogen; CRP, C-reactive protein; HR, heart rate; INR, international normalized ratio; LVEF, left ventricular ejection fraction (normal LVEF, ≥ 50%; decreased LVEF, < 50%); WBC, white blood cells. ^a^ Lactate values at ICU admission.
