# Supplementary material for: Shock indices are associated with in-hospital mortality among patients with septic shock and normal left ventricular ejection fraction
Source: PLoS One. 2024 Mar 12;19(3):e0298617. doi: 10.1371/journal.pone.0298617 (PMC10931483; doi:10.1371/journal.pone.0298617)
Supplement: S3 Table — CRRT, continuous renal replacement therapy; HFNC, high flow nasal cannula; LVEF, left ventricular ejection fraction (normal LVEF, ≥ 50%; decreased LVEF, < 50%); MV, mechanical ventilation; NIV, non-invasive ventilation. (DOCX) [file pone.0298617.s003.docx]

**S3 Table. Completion rates of 3-h sepsis bundle components and ICU treatments between survivors and non-survivors, stratified by LVEF.**

|  |  | Normal LVEF | | | Decreased LVEF | | |
| --- | --- | --- | --- | --- | --- | --- | --- |
|  | Total  (n = 392) | Survivors  (n = 164) | Non-survivors  (n = 82) | P value | Survivors  (n = 71) | Non-survivors  (n = 75) | P value |
| **3-h sepsis bundle components** |  |  |  |  |  |  |  |
| Lactate measurement | 383 (97.7%) | 161 (98.2%) | 79 (96.3%) | 0.381 | 69 (97.2%) | 74 (98.7%) | 0.528 |
| Blood culture | 288 (73.5%) | 115 (70.1%) | 57 (69.5%) | 0.921 | 61 (85.9%) | 55 (73.3%) | 0.060 |
| Antibiotics | 279 (71.2%) | 127 (77.4%) | 56 (68.3%) | 0.121 | 48 (67.6%) | 48 (64.0%) | 0.640 |
| Fluids (any bolus) | 332 (84.7%) | 136 (82.9%) | 70 (85.4%) | 0.625 | 61 (85.9%) | 65 (86.7%) | 0.895 |
| Vasopressors | 339 (86.5%) | 140 (85.4%) | 69 (84.1%) | 0.801 | 61 (85.9%) | 69 (92.0%) | 0.239 |
| **ICU treatments within 3 days** |  |  |  |  |  |  |  |
| Antibiotic adequacy | 343 (87.7%) | 152 (92.7%) | 66 (81.5%) | **0.008** | 62 (87.3%) | 63 (84.0%) | 0.567 |
| MV | 221 (56.4%) | 59 (36.0%) | 61 (74.4%) | **< 0.001** | 36 (50.7%) | 65 (86.7%) | **< 0.001** |
| CRRT | 127 (32,4%) | 34 (20.7%) | 38 (46.3%) | **< 0.001** | 16 (22.5%) | 39 (52.0%) | **< 0.001** |
| NIV | 14 (3.6%) | 6 (3.7%) | 2 (2.4%) | 0.611 | 5 (7.0%) | 1 (1.3%) | 0.109 |
| HFNC | 69 (17.6) | 29 (17.7%) | 14 (17.1%) | 0.906 | 15 (21.1%) | 11 (14.7%) | 0.308 |

CRRT, continuous renal replacement therapy; HFNC, high flow nasal cannula; LVEF, left ventricular ejection fraction (normal LVEF, ≥ 50%; decreased LVEF, < 50%); MV, mechanical ventilation; NIV, non-invasive ventilation.
