# Supplementary material for: Shock indices are associated with in-hospital mortality among patients with septic shock and normal left ventricular ejection fraction
Source: PLoS One. 2024 Mar 12;19(3):e0298617. doi: 10.1371/journal.pone.0298617 (PMC10931483; doi:10.1371/journal.pone.0298617)
Supplement: S4 Table — Age-SI, age shock index; DSI, diastolic shock index; LVEF, left ventricular ejection fraction (normal LVEF, ≥ 50%; decreased LVEF, < 50%); MSI, modified shock index; SI, systolic shock index. a Delta values mean changes from the time zero to ICU admission: negative values indicate a decrease (i.e., improvement) in the shock indices at ICU admission compared to those at time zero. b N = 3. (DOCX) [file pone.0298617.s004.docx]

**S4 Table. Changes in the four shock indices both in patients who completed 3-h sepsis bundle components and in those who did not.**

| 3-h sepsis bundle components | Variables ^a^ | Normal LVEF (n = 246) | | | Decreased LVEF (n = 146) | | |
| --- | --- | --- | --- | --- | --- | --- | --- |
|  |  | Completion (+) | Completion (-) | P value | Completion (+) | Completion (-) | P value |
| Vasopressors | Delta SI | -0.26 (-0.59 to 0.020) | -0.02 (-0.14 to 0.46) | **< 0.001** | -0.09 (--0.45 to 0.27) | 0.05 (-015 to 0.30) | 0.244 |
|  | Delta DSI | -0.29 (-0.84 to 0.20) | 0.25 (-0.12 to 0.70) | **< 0.001** | -0.11 (-0.56 to 0.48) | 0.15 (-0.19 to 0.51) | 0.227 |
|  | Delta MSI | -0.28 (-0.71 to 0.08) | 0.14 (-0.17 to 0.55) | **< 0.001** | -0.18 (-0.51 to 0.43) | 0.06 (-0.17 to 0.36) | 0.246 |
|  | Delta Age-SI | -17.26 (-39.37 to 1.33) | -1.40 (-11.05 to 30.22) | **< 0.001** | -4.62 (-33.26 to 15.12) | 2.55 (-11.14 to 23.54) | 0.240 |
| Fluids | Delta SI | -0.25 (-0.55 to 0.06) | -0.05 (-0.39 to 0.27) | **0.032** | -0.07 (-0.43 to 0.29) | -0.04 (-0.46 to 0.18) | 0.774 |
|  | Delta DSI | -0.23 (-0.81 to 0.25) | -0.03 (-0.47 to 0.53) | 0.087 | -0.05 (-0.57 to 0.51) | -0.06 (-0.47 to 0.34) | 0.989 |
|  | Delta MSI | -0.23 (-0.69 to 0.13) | -0.04 (-0.40 to 0.41) | 0.054 | -0.08 (-0.51 to 0.47) | -0.18 (-0.32 to 0.25) | 0.927 |
|  | Delta Age-SI | -16.57 (-37.76 to 4.69) | -2.89 (-24.68 to 14.77) | **0.032** | -4.57 (-32.21 to 17.28) | -2.76 (-33.02 to 13.52) | 0.943 |
| Antibiotics | Delta SI | -0.22 (-0.45 to 0.07) | -0.22 (-0.63 to 0.18) | 0.928 | -0.11 (-0.46 to 0.21) | -0.08 (-0.28 to 0.31) | 0.129 |
|  | Delta DSI | -0.22 (-0.83 to 0.26) | -0.13 (-0.70 to 0.44) | 0.554 | -0.05 (-0.56 to 0.46) | -0.05 (-0.51 to 0.56) | 0.628 |
|  | Delta MSI | -0.20 (-0.68 to 0.14) | -0.16 (-0.69 to 0.19) | 0.676 | -0.19 (-0.52 to 0.39) | 0.00 (-0.39 to 0.48) | 0.306 |
|  | Delta Age-SI | -15.33 (-33.66 to 4.86) | -14.79 (-45.26 to 10.03) | 0.885 | -7.55 (-33.59 to 11.59) | -5.33 (-19.12 to 20.63) | 0.160 |
| Lactate measurement | Delta SI | -0.22 (-0.52 to 0.08) | 0.27 (-0.15 to 099) | **0.027** | -0.06 (-0.44 to 0.26) | - ^b^ | 0.252 |
|  | Delta DSI | -0.21 (-0.74 to 0.26) | 0.25 (-0.42 to 1.33) | 0.174 | -0.05 (-0.54 to 0.47) | - ^b^ | 0.416 |
|  | Delta MSI | -0.20 (-0.68 to 0.14) | 0.27 (-0.27 to 1.2) | 0.066 | -0.12 (-0.49 to 0.41) | - ^b^ | 0.273 |
|  | Delta Age-SI | -15.40 (-36.45 to 5.42) | 15.27 (-10.00 to 78.02) | **0.031** | -4.05 (-33.06 to 14.76) | - ^b^ | 0.202 |
| Blood culture | Delta SI | - 0.19 (-0.45 to 0.16) | -0.26 (-0.66 to 0.03) | 0.082 | -0.07 (-0.39 to 0.28) | --0.01 (-0.57 to 0.28) | 0.790 |
|  | Delta DSI | -0.19 (-0.74 to 0.30) | -0.23 (-0.73 to 0.14) | 0.318 | -0.08 (-0.56 to 0.46) | 0.04 (-0.51 to 0.53) | 0.697 |
|  | Delta MSI | -0.15 (-0.67 to 0.22) | -0.27 (-0.74 to 0.00) | 0.134 | -0.15 (-0.48 to 0.42) | 0.04 (-0.52 to 0.42) | 0.877 |
|  | Delta Age-SI | -13.64 (-34.64 to 10.00) | -17.00 (-40.43 to -2.29) | 0.112 | -4.31 (-28.65 to 13.78) | -0.51 (-41.00 to 21.17) | 0.879 |

Age-SI, age shock index; DSI, diastolic shock index; LVEF, left ventricular ejection fraction (normal LVEF, ≥ 50%; decreased LVEF, < 50%); MSI, modified shock index; SI, systolic shock index. ^a^ Delta values mean changes from the time zero to ICU admission: negative values indicate a decrease (i.e., improvement) in the shock indices at ICU admission compared to those at time zero. ^b^ N = 3
