# Supplementary material for: Shock indices are associated with in-hospital mortality among patients with septic shock and normal left ventricular ejection fraction
Source: PLoS One. 2024 Mar 12;19(3):e0298617. doi: 10.1371/journal.pone.0298617 (PMC10931483; doi:10.1371/journal.pone.0298617)
Supplement: S5 Table — Age-SI, age shock index; CI, confidence interval; DSI, diastolic shock index; I, input; ICU, intensive care unit; LVEF, left ventricular ejection fraction (normal LVEF, ≥ 50%; decreased LVEF, < 50%); MSI, modified shock index; O, output; SAPS, simplified acute physiology score; SI, systolic shock index; SOFA, sequential organ failure assessment. a Within 3 h of the time zero. (DOCX) [file pone.0298617.s005.docx]

**S5 Table. Univariable analyses of risk factors for in-hospital mortality in patients with normal LVEF (n = 246).**

| Variables | Odd Ratios | 95% CIs | P value |
| --- | --- | --- | --- |
| Age | 1.000 | 0.981 to 1.021 | 0.997 |
| Gender | 1.217 | 0.715 to 2.071 | 0.469 |
| Body mass index | 1.019 | 0.956 to 1.085 | 0.565 |
| Respiratory rate | 1.002 | 0.964 to 1.042 | 0.905 |
| Body temperature | 0.737 | 0.578 to 0.940 | **0.014** |
| SI at ICU admission | 2.963 | 1.482 to 5.922 | **0.002** |
| DSI at ICU admission | 2.183 | 1.423 to 3.347 | **< 0.001** |
| MSI at ICU admission | 2.616 | 1.504 to 4.550 | **0.001** |
| Age SI at ICU admission | 1.011 | 1.002 to 1.019 | **0.014** |
| Chronic heart disease | 1.248 | 0.711 to 2.188 | 0.440 |
| Chronic lung disease | 1.427 | 0.733 to 2.778 | 0.295 |
| Chronic neurological disease | 0.876 | 0.471 to 1.631 | 0.676 |
| Chronic liver disease | 0.484 | 0.189 to 1.240 | 0.131 |
| Diabetes | 0.663 | 0.381 to 1.149 | 0.143 |
| Chronic kidney disease | 1.427 | 0.733 to 2.778 | 0.295 |
| Connective tissue disease | 1.350 | 0.375 to 4.925 | 0.649 |
| Immunocompromised | 3.076 | 0.504 to 18.782 | 0.224 |
| Cancer | 1.945 | 1.084 to 3.490 | **0.026** |
| Charlson comorbidity index | 1.055 | 0.952 to 1.169 | 0.309 |
| SOFA at time zero | 1.071 | 0.984 to 1.165 | 0.112 |
| SOFA total at ICU admission | 1.217 | 1.111 to 1.334 | **< 0.001** |
| SAPS3 at ICU admission | 1.092 | 1.064 to 1.122 | **< 0.001** |
| Hospital-acquired infections | 1.820 | 1.064 to 3.114 | **0.029** |
| Bacteremia | 0.551 | 0.316 to 0.962 | **0.036** |
| Multi-drug resistant pathogens | 0.812 | 0.357 to 1.845 | 0.619 |
| Inappropriate antibiotics | 2.879 | 1.278 to 6.486 | **0.011** |
| Antibiotic administration within 3 h ^a^ | 0.627 | 0.347 to 1.134 | 0.123 |
| Vasopressor use within 3 h ^a^ | 0.910 | 0.437 to 1.896 | 0.801 |
| Fluid bolus within 3 h ^a^ | 1.201 | 0.576 to 2.505 | 0.625 |
| Lactate measurement within 3 h ^a^ | 0.491 | 0.097 to 2.486 | 0.390 |
| Blood culture within 3 h ^a^ | 0.971 | 0.546 to 1.730 | 0.922 |

Age-SI, age shock index; CI, confidence interval; DSI, diastolic shock index; I, input; ICU, intensive care unit; LVEF, left ventricular ejection fraction (normal LVEF, ≥ 50%; decreased LVEF, < 50%); MSI, modified shock index; O, output; SAPS, simplified acute physiology score; SI, systolic shock index; SOFA, sequential organ failure assessment. ^a^ Within 3 h of the time zero.
