# Supplementary material for: Shock indices are associated with in-hospital mortality among patients with septic shock and normal left ventricular ejection fraction
Source: PLoS One. 2024 Mar 12;19(3):e0298617. doi: 10.1371/journal.pone.0298617 (PMC10931483; doi:10.1371/journal.pone.0298617)
Supplement: S6 Table — ICU, intensive care unit; LVEF, left ventricular ejection fraction (normal LVEF, ≥ 50%; decreased LVEF, < 50%); SI, shock index. (DOCX) [file pone.0298617.s006.docx]

**S6 Table. Proportions of patients with SI > 1.0 by LVEF, lactate levels, and in-hospital mortality.**

| Variables | At time zero | At ICU admission |
| --- | --- | --- |
| LV function |  |  |
| Normal LVEF (n = 246) | 186/246 | 131/246 |
| Decreased LVEF (n = 146) | 117/146 | 103/146 |
| Lactate levels |  |  |
| Lactate of ≤ 4 mmol/L | 108/152 | 84/152 |
| Lactate of > 4 mmol/L | 195/240 | 150/240 |
| In-hospital mortality |  |  |
| Survivors | 179/235 | 127/235 |
| Non-survivors | 124/157 | 107/157 |

ICU, intensive care unit; LVEF, left ventricular ejection fraction (normal LVEF, ≥ 50%; decreased LVEF, < 50%); SI, shock index.
