# Supplementary material for: Shock indices are associated with in-hospital mortality among patients with septic shock and normal left ventricular ejection fraction
Source: PLoS One. 2024 Mar 12;19(3):e0298617. doi: 10.1371/journal.pone.0298617 (PMC10931483; doi:10.1371/journal.pone.0298617)
Supplement: S1 Fig — Normal function indicates LVEF of ≥ 50%, and mild, moderate, and severe dysfunctions indicate LVEF of 40–49%, 20–39%, and < 20%, respectively. (DOCX) [file pone.0298617.s007.docx]

**S1 Fig. In-hospital mortality according to left ventricular ejection fraction (LVEF) among patients with septic shock.**


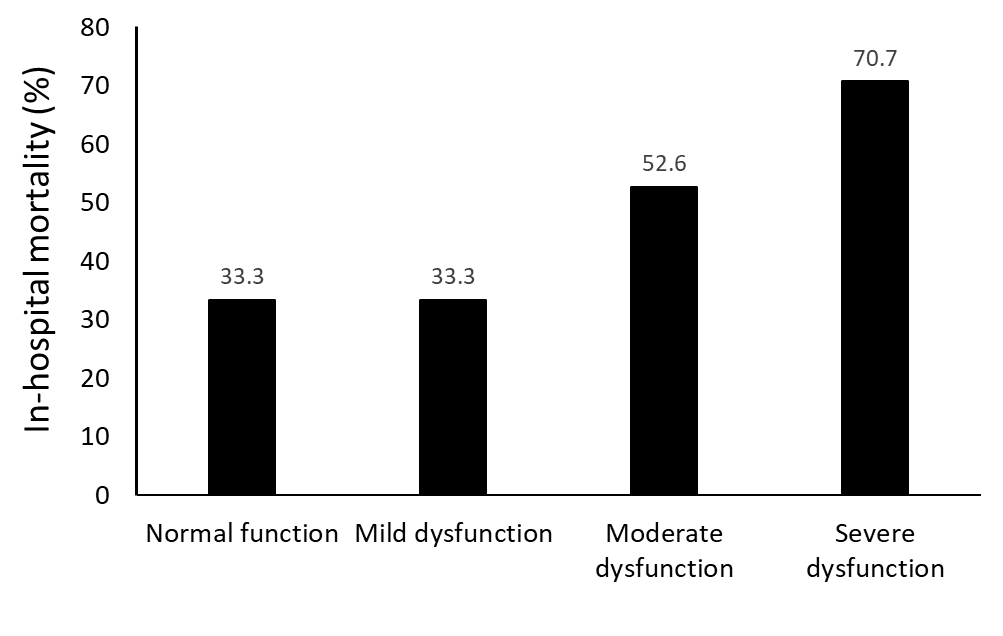


Normal function indicates LVEF of ≥ 50%, and mild, moderate, and severe dysfunctions indicate LVEF of 40 – 49%, 20 – 39%, and < 20%, respectively.
