# Supplementary material for: Shock indices are associated with in-hospital mortality among patients with septic shock and normal left ventricular ejection fraction
Source: PLoS One. 2024 Mar 12;19(3):e0298617. doi: 10.1371/journal.pone.0298617 (PMC10931483; doi:10.1371/journal.pone.0298617)
Supplement: S2 Fig — A) patients with normal or hyperdynamic LVEF, B) patients with decreased LVEF. Age-SI, age shock index, LVEF, left ventricular ejection fraction. (DOCX) [file pone.0298617.s008.docx]

**S2 Fig. Changes in Age-SI from time zero to ICU admission.**

**A)**

**
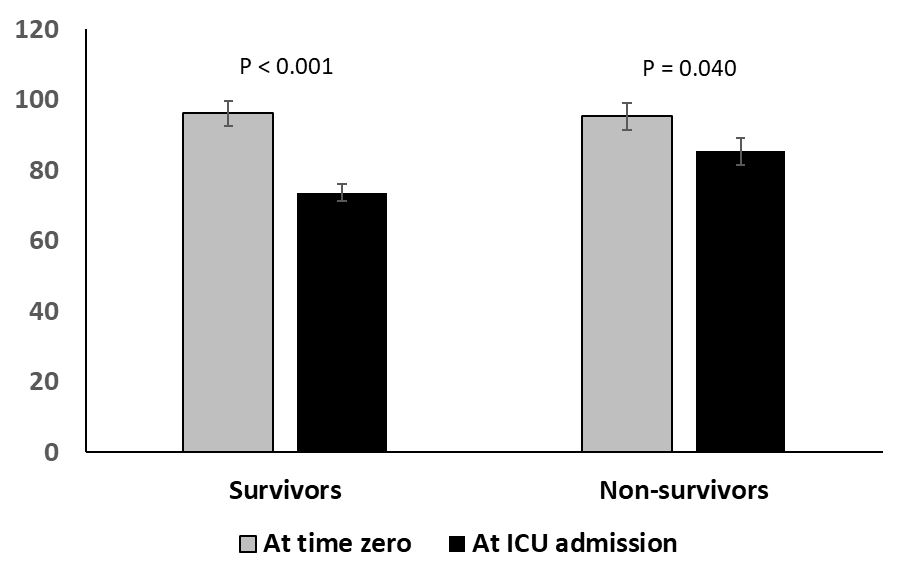
**

**B)**

**
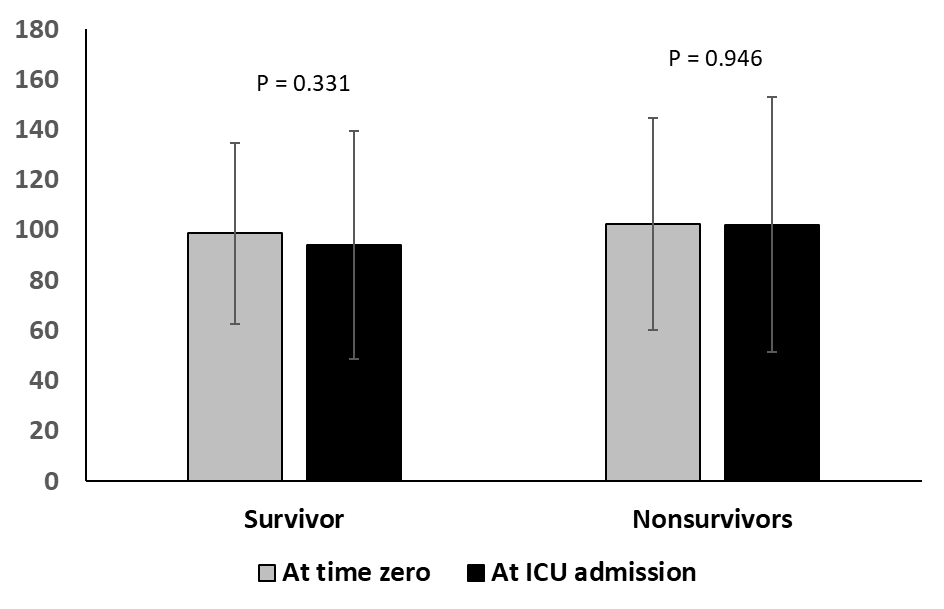
**

A) patients with normal LVEF, B) patients with decreased LVEF. Age-SI, age shock index, LVEF, left ventricular ejection fraction.
