# Supplementary material for: Shock indices are associated with in-hospital mortality among patients with septic shock and normal left ventricular ejection fraction
Source: PLoS One. 2024 Mar 12;19(3):e0298617. doi: 10.1371/journal.pone.0298617 (PMC10931483; doi:10.1371/journal.pone.0298617)
Supplement: S3 Fig — AUC, area under the ROC curve; CI, condifence interval; SBP, systolic blood pressure; DBP, diastolic blood pressure; HR, heart rate; SI, shock index; DSI, diastolic shock index; MSI, modified shock index; Age-SI, age shock index; LVEF, left ventricular ejection fraction. (DOCX) [file pone.0298617.s009.docx]

**S3 Fig. Receiver operating characteristics (ROC) curves for predicting in-hospital mortality among 262 patients with normal LVEF (≥ 50%).**


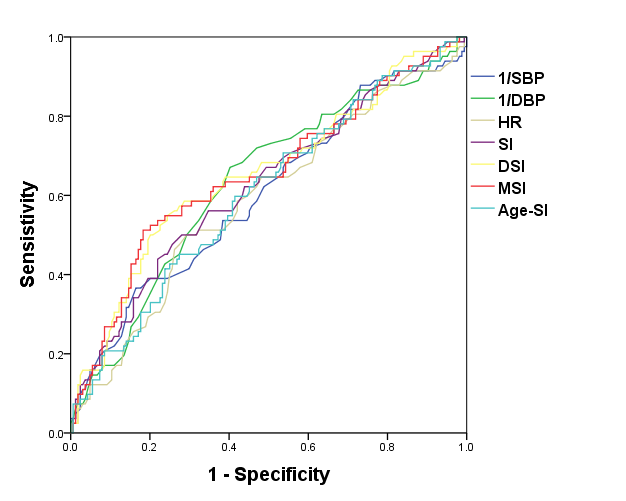


| Variables | AUC | 95% CI | P value |
| --- | --- | --- | --- |
| 1/SBP | 0.604 | 0.527 to 0.680 | **0.008** |
| 1/DBP | 0.633 | 0.559 to 0.708 | **0.001** |
| HR | 0.589 | 0.512 to 0.666 | **0.023** |
| SI | 0.626 | 0.550 to 0.701 | **0.001** |
| DSI | 0.653 | 0.578 to 0.728 | **0.000** |
| MSI | 0.651 | 0.575 to 0.727 | **0.000** |
| Age-SI | 0.605 | 0.530 to 0.680 | **0.007** |

AUC, area under the receiver operating characteristics curve; CI, condifence interval; SBP, systolic blood pressure; DBP, diastolic blood pressure; HR, heart rate; SI, shock index; DSI, diastolic shock index; MSI, modified shock index; Age-SI, age shock index; LVEF, left ventricular ejection fraction.
